# The role of OX40L and ICAM-1 in the stability of coronary atherosclerotic plaques and their relationship with sudden coronary death

**DOI:** 10.1186/s12872-019-1251-8

**Published:** 2019-11-29

**Authors:** Yu Wang, Xiaoyu Sun, Bing Xia, Cuiyun Le, Zhu Li, Jie Wang, Jiang Huang, Jiawen Wang, Changwu Wan

**Affiliations:** School of Forensic Medicine, Guizhou Medical University, Guiyang, Guizhou Province 550004 People’s Republic of China

**Keywords:** Coronary atherosclerosis, Plaque stability, Sudden death, OX40L, ICAM-1

## Abstract

**Background:**

Coronary heart disease is related to sudden death caused by multi-factors and a major threat to human health.This study explores the role of OX40L and ICAM-1 in the stability of coronary plaques and their relationship with sudden coronary death.

**Methods:**

A total of 118 human coronary arteries with different degrees of atherosclerosis and/or sudden coronary death comprised the experimental group and 28 healthy subjects constituted the control group were isolated from patients. The experimental group was subdivided based on whether the cause of death was sudden coronary death and whether it was accompanied by thrombosis, plaque rupture, plaque outflow and other secondary changes: group I: patients with coronary atherosclerosis but not sudden coronary death, group II: sudden coronary death without any of the secondary changes mentioned above, group III: sudden coronary death with coronary artery atherosclerotic lesions accompanied by either of the above secondary changes. The histological structure of the coronary artery was observed under a light microscope after routine HE staining, and the related indexes of atherosclerotic plaque lesions were assessed by image analysis software. The expressions of OX40L and ICAM-1 were detected by real-time quantitative PCR (RT-PCR), immunohistochemistry (IHC) and Western blotting, and the correlations between the expressions and the stability of coronary atherosclerotic plaque and sudden coronary death were analyzed.

**Results:**

(1) The expression of OX40L protein in the control group and the three experimental groups showed an increasing trend, and the difference between groups was statistically significant (*P* < 0.05). (2) The expression of the ICAM-1 protein in the control group and the three experimental groups showed a statistically significant (*P <* 0.05) increasing trend. (3) The expression of OX40L and ICAM-1 mRNAs increased in the control and the three experimental groups and the difference was statistically significant (*P* < 0.05).

**Conclusion:**

The expression of OX40L and ICAM-1 proteins and mRNAs is positively correlated with the stability of coronary atherosclerotic plaque and sudden coronary death.

## Background

Coronary atherosclerosis is a chronic inflammatory disease characterized by the formation of atherosclerotic plaques in the walls of coronary arteries. The development and deterioration of diseases are closely related to the stability of these atherosclerotic plaques. The characteristics of plaque instability include enlargement of necrotic foci, increased number of inflammatory macrophages, and thinning of the fibrous cap. When a plaque develops an unstable phenotype, it might rupture easily, leading to thrombosis and subsequent myocardial infarction, stroke, or sudden death. Despite significant advances in the treatment of heart disease in recent years, the rupture of atherosclerotic plaques remains the leading cause of death due to acute heart diseases. Therefore, investigating the risk factors of atherosclerosis is essential to identify novel therapeutic targets or preventive methods. Previous studies have shown that the occurrence of coronary heart disease (CHD) and sudden death are linked with the stability of coronary atherosclerotic plaque; however, the cause and mechanism of changes in plaque stability are yet to be clarified [1].

OX40 and OX40L are a pair of complementary transmembrane glycoproteins and members of the tumor necrosis factor (TNF) family, which mediates the co-stimulatory signals. The glycoproteins are involved in the occurrence and progression of atherosclerosis and ACS (acute coronary syndrome), which can activate the T cell signaling pathway, participate in the activation, proliferation, and migration of T cells, and maintain the long-term survival of T cells. It is an essential co-stimulatory molecule in the inflammatory immune response of the human body [2]. Moreover, the OX40/OX40L system plays a major role in the overall development of atherosclerosis [3]. It enhances the function of T lymphocytes that promotes the inflammatory responses and accelerate the progression of tissue necrosis, especially of the formation, development, and disruption of unstable plaques [2]. However, these studies only were only focused on the serological detection using animal models and population genetic testing, which provided indirect results [4]. The direct detection of the expression of OX40/OX40L in the human coronary vascular tissue has not yet been reported.

ICAM-1 is a member of the immunoglobulin superfamily. It is seldom expressed or not expressed under normal conditions. However, ICAM-1 is widely expressed in a variety of cells after stimulation of the inflammatory factors, which enhances the adhesion between cells and vascular endothelium, mediates the inflammatory cells such as monocytes into vascular endothelium, and promotes the occurrence, development, and deterioration of AS. Oishi et al. found that increased serum ICAM-1 levels were associated with the progression of coronary atherosclerosis, and hence, could be used as a marker of the severity of coronary atherosclerosis [5]. Haim et al. followed up 136 patients with coronary artery disease for 6.2 years. The results showed that the probability of coronary artery events increased by 11.27% with the increase in the level of ICAM-1 to 100 μg/L. The higher the level of ICAM-1, the greater the probability of coronary artery events [6].

The aim of the present study was to detect the expression of OX40L and ICAM-1 in the atherosclerotic plaque by collecting the coronary artery. The correlation between the structural stability of atherosclerotic plaque and the sudden death due to the CHD was analyzed to provide sufficient evidence for the forensic identification and the experimental basis for the prevention and treatment of the disease.

## Methods

### Authentic samples

This study was approved by the Ethics Committee of Guizhou Medical University (Lot No.: 2018 Renxu No. 01). The specimens of cardiac coronary arteries were obtained by autopsy at the Forensic Center of Guizhou Medical University, from January 2014 to March 2017. The inclusion criteria of the research subjects were as follows: (1) The frozen corpse was dissected within 7 days, and the body was not frozen or refrigerated for 48 h; (2) The coronary atherosclerosis in the experimental group was clearly confirmed by the anatomical and histological examinations; (3) Simultaneous anatomical and histological examinations confirmed that cases without coronary atherosclerosis were included in the control group.

The exclusion criteria of the subjects: (1) The tissue from the deceased has been corrupted or autolyzed; (2) The deceased had cachexia or multiple organ dysfunction disorders; (3) The deceased had sepsis or other infectious inflammatory diseases.

According to the above criteria, samples of coronary artery vascular tissue were collected from 146 cases. A portion of each sample was immersed in 4% neutral formalin for routine hematoxylin-eosin (HE) and IHC, and the remaining was preserved at − 80 °C for Western blotting and real-time fluorescent quantitative PCR.

### Experimental grouping

All specimens were divided into experimental and control groups. In the experimental group, the stenotic vessels in the coronary artery were examined visually, and the anterior descending branch of the coronary artery was examined in the control group. Based on the coronary artery lesion and the sudden death in patients with coronary heart disease, the experimental group was divided into three groups. Group I: atherosclerosis but no sudden coronary death (SCD); group II: sudden coronary death but coronary atherosclerotic lesions without thrombosis, plaque block rupture, entablature hemorrhage, and other secondary lesions; group III: sudden coronary death and coronary atherosclerotic lesions associated with any of the above secondary lesions.

### Histopathological observation of blood vessels and morphological metrology analysis

The coronary artery was fixed with 4% neutral formaldehyde, embedded in conventional paraffin, sectioned, and subjected to HE staining to observe the structural changes of the coronary atherosclerotic plaques. A cross-section of the whole blood vessel was obtained. IPP 6.0 image analysis software was used to detect the related morphological indexes.

1. Intimal and lesion thicknesses: From the free edge of the endocardial cavity surface to the vertical distance of the internal elastic membrane, the thickest and thinnest thickness of the intima was measured, and another straight line was perpendicular to the two test lines. The mean of the test line and the average thickness of the intima were calculated.
2. Thickness of the fibrous cap: The fibrous cap on the surface of atherosclerotic lesions of coronary arteries was assessed in the experimental group. The thickness of the fibrous cap on both sides of the cap and at the thickest intimal was measured, and the average value was obtained.
3. Thickness of necrosis: The vertical distance from the proximal rim to the proximal margin, the largest and smallest necrosis lesions in atherosclerosis were evaluated, and another test line was drawn perpendicular to the test line, with three lines in total. The mean value was the average thickness of the necrotic lesions, while necrosis was not evaluated.
4. Degree of lumen stenosis: The ratio of intimal area to the sum of intima and lumen area was assessed.

### Expression and distribution of OX40L and ICAM-1 proteins in coronary artery lesions

The expression of OX40L and ICAM-1 proteins was observed by IHC using PV two-step method. The above paraffin-embedded tissues were sectioned into 4 μm, deparaffinized, and hydrated. 3% hydrogen peroxide was used to eliminate the endogenous peroxidase, and high-pressure heat retrieved the antigen epitope, followed by incubating the sections overnight at 4 °C with OX40L murine monoclonal antibody (1:100; Abcam, UK) and ICAM-1 murine monoclonal antibody (1:75; GeneTex, USA), while PBS buffer was used as the negative control and incubated. Subsequently, the sections were incubated with horseradish peroxidase-conjugated goat anti- murine/rabbit IgG (PV9000 two-step kit, ZSGB-BIO, China) at room temperature for 40 min. Finally, the sections were visualized with DAB for 2 min, countersigned with hematoxylin for 30 s, and observed under a light microscope. The positive expression of OX40L and ICAM-1 was detected by the brownish yellow particles on the cell membrane. After observing the type, location, and intensity of the positively-expressing cells under a microscope, five fields of view (FOV) on the shoulders, basement, fibrous cap area, and the peripheral area of the necrotic coronary lesions were photographed at 400× magnification using a microscope. The average optical density value (positive expression optical density value/measured total area) was measured with respect to the level of the reactive protein expression.

### Determination of the expression of OX40L and ICAM-1 proteins

The expression of OX40L and ICAM-1 in the coronary artery was detected by Western blot semi-quantitative method. An equivalent of 70–90 mg frozen cryopreserved coronary vascular specimens were homogenized in 1 mL of protein lysis buffer (RIPA+PMSF protease inhibitors; Beyotime, China) to obtain the protein extract. The concentration of the protein extract was measured by an ultraviolet spectrophotometer, and β-actin served as an internal reference. The extracted protein was separated on 10% SDS-PAGE, followed by transfer to PVDF membrane. Then, the membrane was blocked with 5% skim milk, followed by probing with primary antibodies at 4 °C overnight: β-actin murine monoclonal antibody (1:2000; Abbkine, USA), OX40L rabbit monoclonal antibody (1:10000; Abcam, and ICAM-1 murine monoclonal antibody (1:1000; GeneTex). Subsequently, the membrane was incubated with horseradish peroxidase-conjugated goat anti- murine IgG (1:8000; Solarbio, China) and goat anti-rabbit IgG (1:10000; ThermoFisher Scientific, USA). The immunoreactive bands were visualized, and the Image J analysis software was used to scan the total gray value of each band. The ratio of the total gray value of the target band was normalized to that of the β-actin.

### Determination of mRNA expression of OX40L and ICAM-1

The total RNA of the cryopreserved human coronary vascular tissue (weight 70–90 mg) was extracted using TRIzol (Invitrogen, USA) reagent according to the manufacturer’s instructions. The concentration of RNA was measured using a UV spectrophotometer and reverse transcribed according to the manufacturer’s protocol (PrimeScript™ RT reagent Kit, Japan). After the addition of SYBR® Select MasterMix reagent (Applied Biosystems, USA) and the corresponding primers, CT values were measured on a 7500 Fast real-time PCR instruments (Applied Biosystems). The level of the target gene transcription was calculated according to the manufacturer’s method, using the 2^-ΔΔCt^ formula. The PCR thermocycling conditions were as follows: 50 °C for 2 min, 95 °C for 2 min, 40 cycles with 95 °C for 30 s, 60 °C for 30 s, and 72 °C for 1 min. Dissociation curve conditions were as follows: 95 °C for 15 s, 60 °C for 1 min, 95 °C for 15 s. The primers were searched on GenBank NCBI, designed using BLAST, synthesized by Shanghai Bioengineering Co., Ltd. (China). The primer sequences were as follows: murine β-actin: F: CATCATGAAGTGTGACGTGG, R: TCGTCATACTCCTGCTTGCT; murine OX40L F: CTGGGACAGAAGGAAAGCTG, R: TGGGAAGTGAGGATGAAACC; murine ICAM-1: F: GGCTGGAGCTGTTTGAGAAC, R: AGGAGTCGTTGCCATAGGTG.

### Statistical analysis

The acquired data and measurement data were expressed as mean ± standard deviation (SD); the mean among the groups was compared using the one way ANOVA test; the homogeneity of variance was detected using the LSD method, and the variance was invariant using the Games–Howell method. The correlation analysis between the indices and the normal distribution were carried out by Pearson’s test. The product-moment correlation coefficient test, which does not obey any normal distribution, was assessed using the Spearman’s rank correlation coefficient. The above results were analyzed by SPSS 22.0 software, and the difference was statistically significant (*P* < 0. 05).

## Results

### Baseline information about the patients

Samples from 86 cases of coronary vascular tissue were collected in the experimental group according to the inclusion and exclusion criteria for morphological observation and analysis, and 32 cases were used for protein quantification and mRNA detection. The experimental group included 88 males and 30 females with an age range from 30 to 83 years (average age, 54.37 ± 13.1 years). The peak age of onset of the disease was 32–60 years. A total of 20/28 patients in the control group were used for morphological observation, while protein and mRNA were quantified in 8 patients. The cohort included 20 males and 8 females with an age range 24–49 years (average 38.11 ± 6.99 years).

### Pathological changes in the coronary arteries

**Fig. 1:**
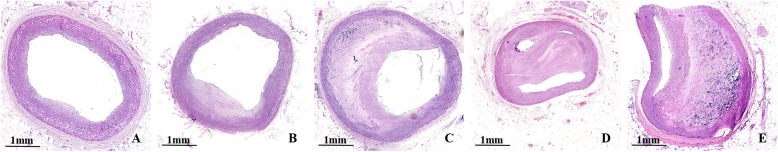
Microscopic changes of coronary atherosclerosis (HE staining, 100×). **a** is the control group, **b** is the experimental group I, **c** is the experimental group II, and **d**, **e** are the experimental group III (thrombosis)

The light microscopy of HE slices showed that the control group had a thin blood vessel wall, uniform thickness, smooth and intact intima, and uniform thickness of the intima, media, and adventitia. In the experimental group I, local thickening of the vessel wall and mild lumen were observed. In the stenosis, foam cells proliferated and aggregated under the endothelium, and irregular infiltration of lymphocytes and other inflammatory cells were observed. In the experimental group II, the thickness of the blood vessel wall increased significantly, and the degree of lumen stenosis was heavier than that of group I. The vascular lesions increased, and typical fibrous caps were regarded. In some cases, atheromatous necrosis was observed under the fibrous cap. Foam cells and granulation were perceived at the bottom and periphery of the necrotic lesions. The tissue hyperplasia and lymphocytic infiltration, mild pressure on the medial atrophy, and changes in the outer membrane were not distinct. In the experimental group III, significant thickening of the vessel wall, severe stenosis of the vessel lumen, and typical atherosclerotic necrosis, i.e., a large amount of amorphous necrosis, cholesterol crystals, and calcification in the lesions, fibrous caps on the lesion surface, and necrosis lesions around the number of residual foam cells and inflammatory cells were varied. In some cases, entablature hemorrhage was detected in the lesions, while in other cases, thrombus formation was observed on the lesion surface. In addition, the rupture of the elastic fibers of the blood vessels, the atrophy and thinning of the smooth muscle cells, the connective tissue hyperplasia, and the lymphocyte and plasma cell infiltration were found on the outer membrane (Fig. 1).

### Morphometric analysis of changes in coronary artery structure

The results of image analysis showed that the thickness of coronary artery intima, the thickness of fibrous cap, the thickness of necrotic foci, and the degree of lumen stenosis in the experimental group was significantly (*P* < 0.05) higher than that in the control group, and the difference was statistically significant. Compared to the experimental group II, the thickness of the fibrous cap in the experimental group III increased gradually with the increase in the area of the necrotic lesion, but the thickness of the fibrous cap did not increase significantly. Compared to the experimental group I, the degree of stenosis in group II increased slightly but not significantly (*P* > 0.05). The comparison between the above indicators in each group was statistically significant (*P* < 0.05) (Table 1).

**Table 1:** Comparison of morphological indexes of coronary artery lesion

Grading	*n*	Intimal thickness (mm)	Fiber cap thickness (mm)	Necrotic lesion thickness (mm)	Degree of lumen stenosis (%)
Control	20	1.13 ± 0.42	0.00 ± 0.00	0.00 ± 0.00	0.00 ± 0.00
Experimental I	26	4.14 ± 0.70^a^	1.90 ± 0.56^a^	3.03 ± 0.76^a^	43.97 ± 0.07^a^
Experimental II	30	5.84 ± 0.40^ab^	3.54 ± 0.62^ab^	5.19 ± 0.43^ab^	63.75 ± 0.11^a^
Experimental III	30	8.76 ± 1.54^abc^	5.03 ± 0.63^ab^	8.07 ± 1.48^abc^	82.31 ± 0.10^abc^

^a^is the comparison with the control group, *P* < 0.05; ^b^is the comparison with the experimental group I, *P* < 0.05; ^c^is the comparison with the experimental group II, *P* < 0.05

### Expression of OX40L and ICAM-1 s protein in coronary atherosclerotic lesions

**Fig. 2:**
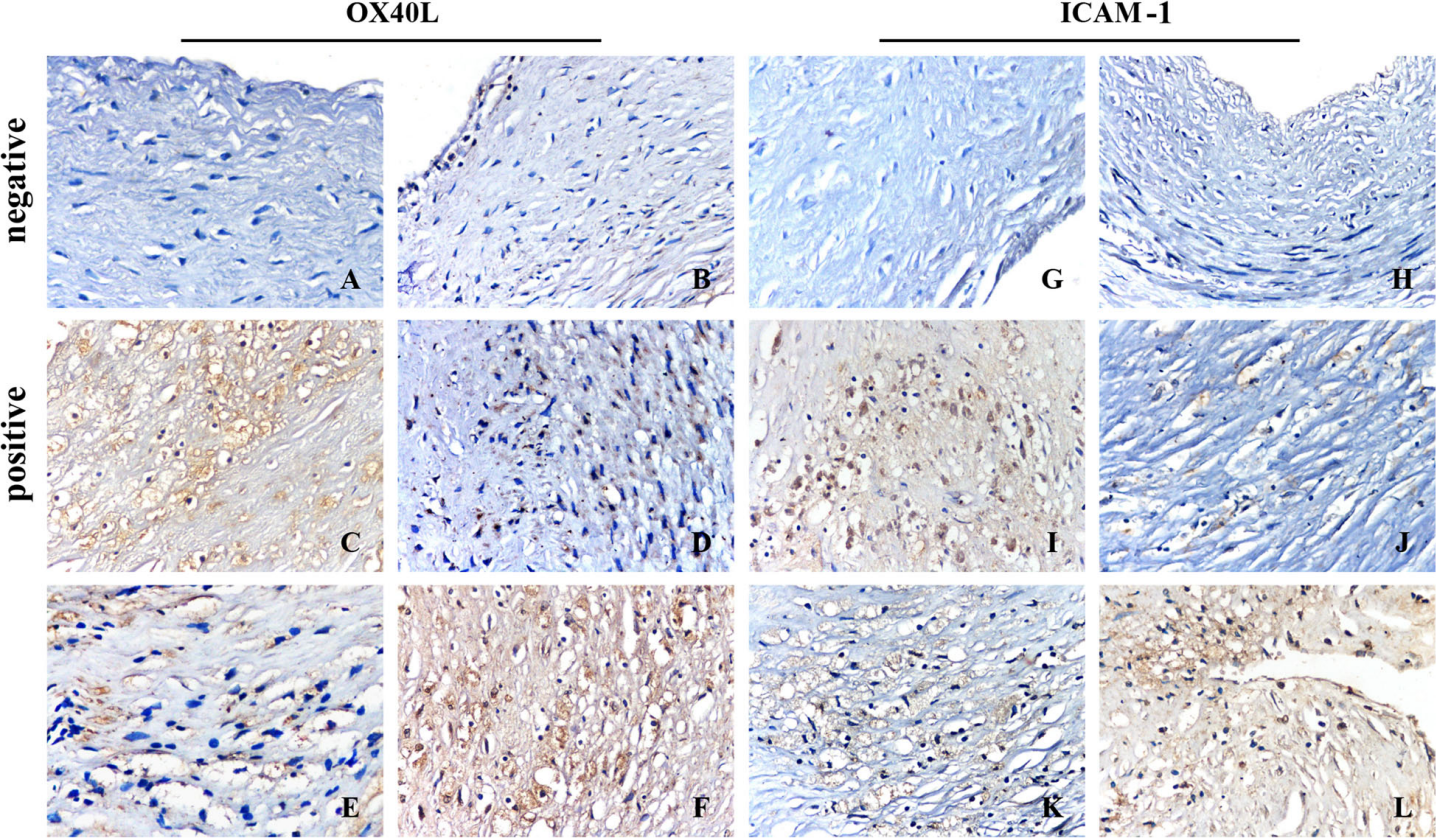
Expression of OX40L and ICAM-1proteins in coronary atherosclerosis (IHC, 400×). **f** is OX40L protein, G-L is ICAM-1 protein; **a** and **g** are negative controls for OX40L and ICAM-1; **c** and **i** are positive controls for OX40L and ICAM-1. **b**, **d**, **e** and **f** are the control, experimental group I, experimental group II and experimental group III of OX40L. **h**, **j**, **k** and **l** are the control, experimental group I, experimental group II and experimental group III of ICAM-1

The expression of OX40L protein was not noted in the vascular wall of the control group. In the experimental group I, the positive expression of OX40L protein showed brownish staining on the surface of the lesion in a few cells. The cells with positive expression occurred in two forms; the large cells were foaming-like cells. Experimental group II showed irregularly distributed brownish-yellow colored cells around the necrotic lesions, primarily in the shoulder and the bottom of the lesion. Furthermore, the experimental group III demonstrated a large number of foam cells on the shoulder and bottom of the lesion. Lymphocytes exhibit a brownish-yellow color on the cell membrane, mainly distributed irregularly around the necrotic foci. The average optical density of OX40L protein expression in the blood vessels was analyzed by IPP6.0 software. Compared to the control group, the positive expression of OX40L in the experimental group was significantly (*P* < 0.05) higher than that in the control group (Fig. 2,Table 2).

**Table 2:** Optical density of expression of OX40L and ICAM-1 proteins in coronary artery lesions

Grading	*n*	OX40L	ICAM-1
Control	20	0.0003 ± 0.0002	0.0003 ± 0.0002
Experimental I	26	0.0030 ± 0.0008	0.0031 ± 0.0039
Experimental II	30	0.0118 ± 0.0011^ab^	0.0122 ± 0.0012^ab^
Experimental III	30	0.0466 ± 0.0107^abc^	0.0518 ± 0.0083^abc^

^a^is the comparison with the control group, *P* < 0.05; ^b^is the comparison with the experimental group I, *P* < 0.05; ^c^is the comparison with the experimental group II, *P* < 0.05

As some slices were dropped during HE staining, the number of samples was counted in 108 cases (Table 2 was the same)

The expression of the ICAM-1 protein was not observed in the vascular wall of the control group. In the experimental group I, the persistent expression of ICAM-1 was indicated by the brownish-yellow staining on the cell membrane of a large number of foam cells. In the experimental group II, the positively stained cells increased gradually increased in the shoulder and bottom of the lesion. In the experimental group III, a large number of foam cells were observed in the shoulder area of the lesion with a weak fibrous cap and around the necrotic lesion, and brownish yellow staining was found on the lymphocyte membrane. The average optical density of the ICAM-1 protein in blood vessels was analyzed by IPP6.0 software. Compared to the control group, the average optical density of ICAM-1 protein in the experimental group III was significantly higher (*P* < 0.05; Fig. 2, Table 2).

### Expression of OX40L and ICAM-1 proteins in coronary artery tissue

**Fig. 3:**
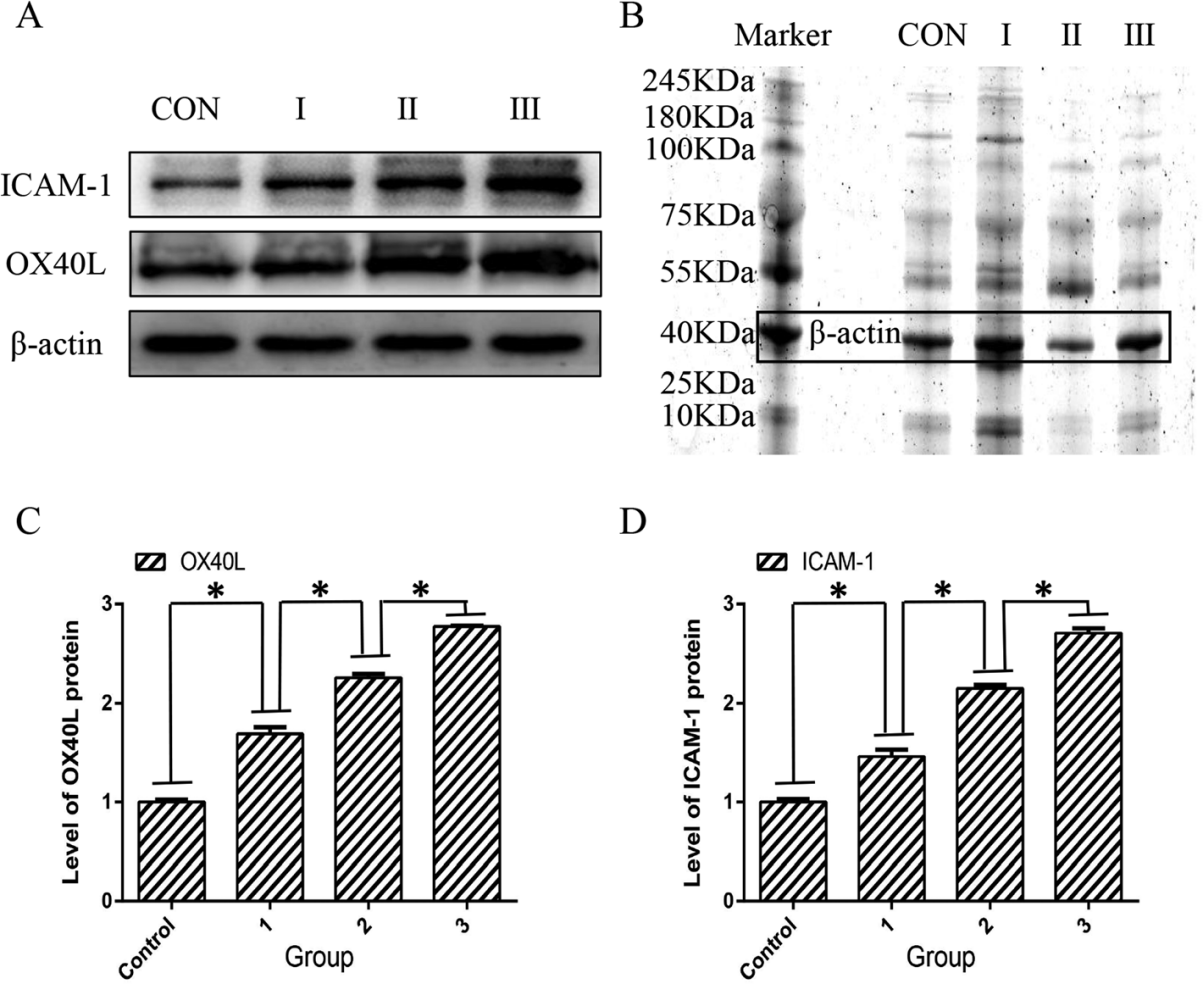
Expression of OX40L and ICAM-1 proteins in coronary atherosclerosis. **a** shows the target protein and internal reference bands. **b** shows the internal reference electropherogram of total protein. **c** is the protein expression level of OX40L. **d** is the protein expression level of ICAM-1. CON and control are control groups. I and 1 indicate experimental group I, II and 2 indicate experimental group II, and III and 3 indicate experimental group III. *p < 0.05 vs. among the groups

Western blotting results showed that the expression of OX40L protein was significantly (*P* < 0.05) increased in the experimental group as compared to the control group. Also, the expression in group III was significantly (*P* < 0.05) higher than that in the experimental group II (Fig. 3).

Compared with the control group, the expression of ICAM-1 protein in the coronary artery tissue of the experimental group increased significantly, and that in experimental groups II and III was significantly higher as compared to the group I(*P* < 0.05). (Fig. 3).

### Expression levels of OX40L and ICAM-1 mRNAs in coronary artery tissue

**Fig. 4:**
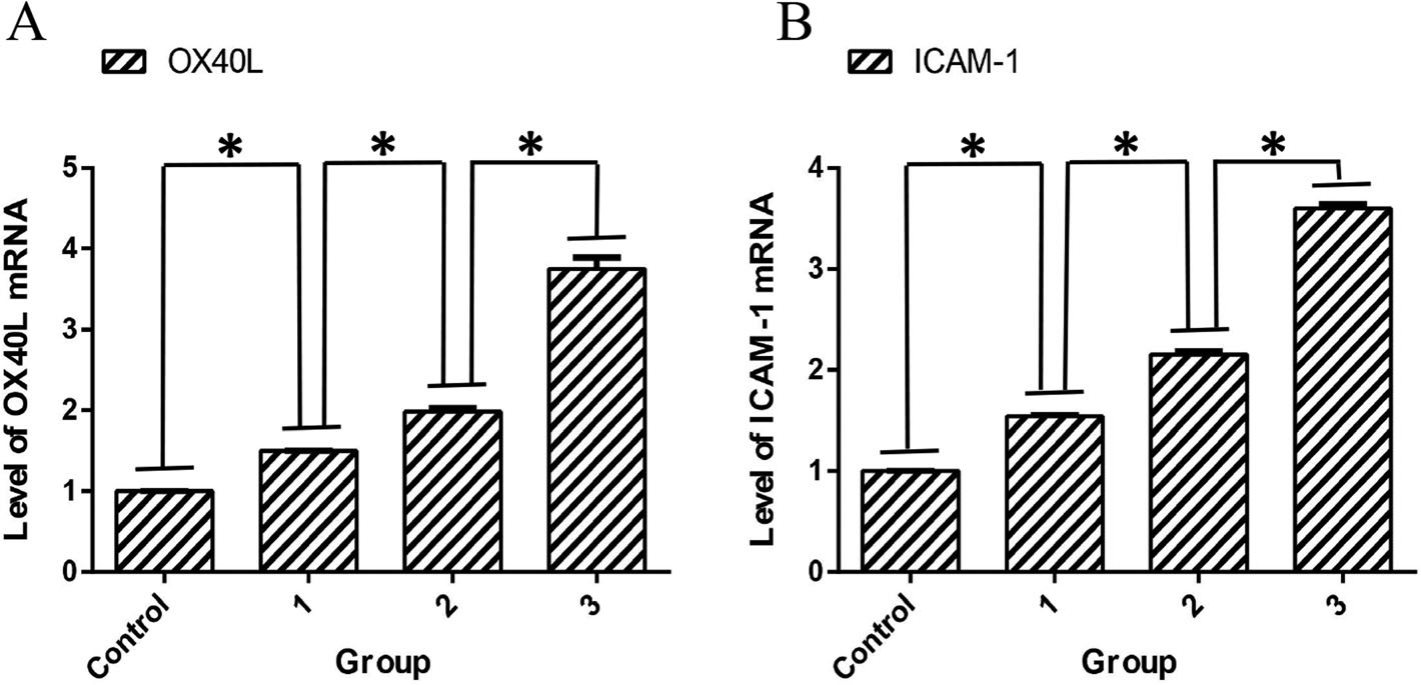
Expression of OX40L and ICAM-1 mRNA in coronary atherosclerosis. **a** is the mRNA expression level of OX40L. **b** is the mRNA expression level of ICAM-1. Control is the control group, 1 is the experimental group I, 2 is the experimental group II, and 3 is the experimental group III.*p < 0.05 vs. among the groups

The results of real-time fluorescence quantitative PCR showed that compared to the control group, the expression levels of OX40L and ICAM-1 mRNA in the experimental group increased significantly (*P* < 0.05). The experimental group was compared between each subgroup, and significant differences were observed (*P* < 0.05; Fig. 4).

### Correlation analysis of morphological OX40L and ICAM-1 proteins’ expression in coronary atherosclerotic lesions

A positive correlation was established between the expression of OX40L and ICAM-1 in coronary vessels. Also, a positive correlation was established between the mean optical density of OX40L and ICAM-1 expression in coronary atherosclerotic lesions (*r* = 0.917,*P* = 0.000), as well as between OX40L and ICAM-1 protein expression in the coronary vascular tissue *(r* = 0.994*, P* = 0.000*)*.

Positive correlation was established between the expression of OX40L and intimal thickness(*r* = 0.833, *P* = 0.000)and thickness of necrotic(*r =* 0.686, *P* = 0.000) in coronary artery lesions, as well as between the expression of ICAM-1 and intimal thickness(*r =* 0.810, *P =* 0.000) and thickness of necrotic(*r =* 0.714,*P =* 0.000)in coronary artery lesions. Negative correlation between OX40L proteins’ expression and the thickness of fibrous cap(*r =* − 0.662*,P =* 0.000). However, the expression of OX40L protein in coronary artery lesions was not correlated with the degree of lumen stenosis (*P =* 0.124).Negative correlation was established between the expression of ICAM-1 and the thickness of fibrous cap(*r =* − 0.353,*P =* 0.000), and the expression of ICAM-1 protein in coronary artery lesions was not correlated with the degree of lumen stenosis (*P =* 0.145).

### Correlation analysis of OX40L and ICAM-1 proteins’ expression in AS lesions and sudden coronary death

Correlation analysis of OX40L proteins’ expression and structural parameters in atheromatous lesions was performed in sudden death cases. The expression of OX40L and ICAM-1were positively correlated with intimal thickness, the thickness of necrotic and the degree of lumen stenosis, negatively correlated with the thickness of fibrous cap(Table 3). Compared the expression levels of OX40L and ICAM-1 in the AS lesions, the levels of OX40L and ICAM-1 in the AS lesions of sudden coronary death were significantly higher than that in the non-sudden coronary death(Table 4).

**Table 3:** Correlation analysis of protein expression level and focal structure parameters

Parameters (SCD cases)	Intimal thickness		Necrotic lesion thickness		Fiber cap thickness		Degree of lumen stenosis	
	*r*	*P*	*r*	*P*	*r*	*P*	*r*	*P*
OX40L	0.796	0.000	0.728	0.000	−0.305	0.021	0.623	0.002
ICAM-1	0.879	0.000	0.603	0.021	−0.268	0.044	0.053	0.005

SCD cases are cases of sudden coronary death; *r* is the correlation coefficient, *P* < 0.05

**Table 4:** Analysis of the protein expression level in the lesions of the SCD and the Non-SCD

parameters	SCD	Non-SCD	*t*	*P*
OX40L	0.0291 ± 0.0191	0.0019 ± 0.0015	27.125	0.001
ICAM-1	0.0320 ± 0.0208	0.0019 ± 0.0033	31.216	0.000

SCD are cases of sudden coronary death, Non-SCD are non-sudden coronary death cases

## Discussion

Coronary artery atherosclerotic heart disease (CAHD) is a heart disease caused by coronary artery atherosclerosis, which leads to coronary artery stenosis or obstruction, myocardial ischemia, hypoxia, or necrosis. It is also known as CHD and often leads to sudden death. Plaque stability is a major factor affecting the acute attack of CHD. Unstable plaque, also known as vulnerable plaque, easily leads to plaque rupture, secondary thrombosis, and other adverse consequences. In addition, the unstable plaque is a major cause of the secondary acute coronary syndrome, which can cause acute myocardial infarction and sudden death [7].

The current studies have shown that inflammation is a critical factor in atherosclerosis, and some inflammatory markers are closely related to atherosclerotic diseases [8]. Blood-derived inflammatory mediators cause arterial endothelial injury and induce smooth muscle cell proliferation. Therefore, plasma levels of inflammatory mediators have been used as markers of arterial wall injury as well as predicting the risk of coronary heart disease [9]. However, previous studies mostly focused on the animal models or inflammatory factors in the blood [10]. The direct detection of inflammatory factors in atherosclerotic plaques revealed a rare correlation between the severity and risk of coronary atherosclerotic plaques [11]. In recent years, the mechanism underlying molecular adhesion and the role of inflammatory immunology in the pathogenesis of atherosclerosis have been under intensive focus [12, 13]. Cell adhesion is the molecular basis for the initiation and acceleration of atherosclerosis, and a major mechanism underlying the formation and development of CHD. ICAM-1 belongs to the immunoglobulin superfamily. It is composed of five Ig-like functional areas, rarely expressed under normal conditions, but widely expressed in a variety of cells under the stimulation of inflammatory factors. It enhances the adhesion between the cells and vascular endothelium and mediates the inflammatory cells such as monocytes into vascular endothelium. In addition, it can promote the occurrence, development, and deterioration of atherosclerosis. Recent studies have shown that ICAM-1, a vital inflammatory marker in vivo, participates not only in the occurrence of inflammatory reaction but also in lipid metabolism and glucose metabolism. However, whether ICAM-1 is related to the severity of coronary artery disease is yet controversial. Santos et al. did not detect any significant difference between ICAM-1 serum concentration and coronary artery lesions [14]. Galkina and Soto have demonstrated an active role of ICAM-1 in the formation of atherosclerotic plaques [10, 15]. Ma et al. found that serum ICAM-1 levels were associated with the clinical classification of CHD, but not with the degree and severity of coronary artery stenosis. This phenomenon suggested that ICAM-1 may be involved in the instability of atherosclerotic plaque, which reflects the severe risk of CHD [16].

IHC showed that ICAM-1 protein was expressed in the endothelial cells of coronary atherosclerotic plaques, especially in foam cells and lymphocytes in atherosclerotic plaques. However, no expression was detected in normal coronary artery walls, which was consistent with previous studies [17]. This study also showed that the expression of foam cells and lymphocytes in unstable plaques was more obvious than that in stable atherosclerotic plaques. The expression of ICAM-1 protein and mRNA in atherosclerotic plaques was more significant than that in the non-CHD patients, indicating it was associated with the stability and crown of coronary atherosclerotic plaques. The risk of heart disease was positively correlated; however, no related studies were reported [18]. The present study suggested that the expression of ICAM-1 protein and mRNA in coronary atherosclerotic plaques may provide a reference for sudden coronary death in forensic pathological practice.

OX40L is the ligand of OX40 and promotes the proliferation and differentiation of T cells by binding with the OX40 receptor. OX40/OX40L regulates the antigen presenting function of macrophages. Recent studies demonstrated a major important role of OX40/OX40L in atherosclerosis. The interaction between OX40L and OX40 promotes the proliferation, differentiation, and survival of T cells and prevents the development of Tregs. The OX40-OX40L interaction is considered to be a potential therapeutic target for autoimmune therapy. The costimulatory OX40-OX40L pathway is crucial in cardiovascular diseases [19]. Single nucleotide polymorphisms in OX40 and OX40L genes have been shown to be closely related to the incidence of cardiovascular disease in humans [20]. The elevated levels of OX40^+^ T cells were found in circulating blood and atherosclerotic plaques in cardiovascular patients [21, 22]. In addition, the levels of serum soluble OX40L were positively correlated with carotid intima-media thickness and serum C-reactive protein levels [23, 24]. Furthermore, animal experiments showed that the incidence of atherosclerotic lesions in OX40L-deficient mice was lower than that in wild-type mice, while the development of atherosclerotic lesions was promoted in the OX40L-overexpressed mice [25]. In addition, the interruption of OX40-OX40L interactions by blocking the antibodies with OX40L resulted in a 53% reduction in the development of atherosclerotic lesions [26]. Another study suggested that blocking the OX40-OX40L interaction in combination with lipid-lowering therapy induced the regression of atherosclerosis [2, 27]. However, any study has not yet reported the correlation between the expression of OX40L protein and mRNA and the stability of atherosclerotic plaques and the risk of sudden coronary death in human cadaveric atherosclerotic plaques.

IHC staining showed that OX40L protein was expressed in foam cells and lymphocytes in stable atherosclerotic plaques than in unstable plaques. Western blotting and real-time fluorescence quantitative PCR showed that the expression of OX40L protein and mRNA in coronary atherosclerotic plaque was higher than that in the non-coronary sudden death. The expression of OX40L protein and mRNA was positively correlated with the stability of coronary atherosclerotic plaque and coronary heart disease, suggesting that the presence of OX40 in coronary atherosclerotic plaque was positively correlated with the expression of OX40L protein and mRNA which provided an in-depth insight into the sudden death of patients with CHD.

The results showed that the average optical density of OX40L in coronary atherosclerotic lesions was positively correlated with that of ICAM-1 (*r* = 0.917, *P* = 0.000), and the expression of OX40L protein in coronary artery was positively correlated with that of ICAM-1 protein (*r* = 0.994, *P* = 0.000). ICAM-1 is the member of the transmembrane glycoprotein family that allows the transmigration of the leukocytes into the vascular intima. The four major steps of leukocyte adhesion cascade by CAMs involve 1) capturing, 2) rolling, 3) adhesion, and 4) transmigration. ICAM-1 “adheres” to leukocyte integrin, which aids in leukocytes migration to the damaged intima, leading to atherosclerosis [28]. OX40L is found on antigen presenting cells, activated T cells, and others including lymphoid tissue inducer cells, endothelia, and mast cells. The expression of both molecules is increased after antigen presentation and also in response to other proinflammatory factors, such as CD28 ligation, CD40L ligation, and IFN-γ signaling. These interactions promote T cell survival, promote an effector T cell phenotype and T cell memory, tend to reduce the regulatory function, increase the effector cytokine production, and enhance the cell mobility [27]. The present study showed a synergistic role of ICAM-1 and OX40L in promoting coronary atherosclerotic plaque instability and sudden death.

Nevertheless, the present study had some limitations. First, the small sample size may have some effect on the result. Second, the protein degrades after death. Although previous studies have shown that cell antigens can be well preserved for 3 days after human death, the inclusion criteria of this study were limited to non-frozen cadavers within 48 h post-death or frozen cadavers within 7 days of autopsy; thus, experimental errors were inevitable [11]. Nevertheless, we used cadaveric coronary arteries to study the correlation between ICAM-1 and OX40L and the stability and risk of coronary atherosclerotic plaques. To the best of our knowledge, this is the first time that a similar study has been carried out. The present study found that ICAM-1 and OX40L play a synergistic role with a positive correlation with coronary atherosclerotic plaque instability and the risk of sudden death events, thereby providing a new reference for the clinical prevention and treatment of CHD and identification of the sudden causes of death in patients.

## Conclusions

In summary, the increase of OX40L and ICAM-1 expression can enhance the inflammatory response in the lesion, destroy the structure of the atherosclerotic plaque and thin the fibrous cap, which is negatively correlated with the thickness of the fibrous cap, and positively correlated with the thickness of the intimal and necrosis. The expression of OX40L and ICAM-1 proteins and mRNAs is positively correlated with the stability of coronary atherosclerotic plaque, this reduces the stability of atheromatous plaques and causes sudden death.

## Data Availability

The datasets used and/or analysed during the current study are available from the corresponding author on reasonable request.

## Abbreviations

ACS: Acute coronary syndrome
CAHD: Coronary artery atherosclerotic heart disease
CHD: Coronary heart disease
SCD: Sudden coronary death
SD: Standard deviation
TNF: Tumor necrosis factor
